# Association of Spike-Specific T Cells With Relative Protection From Subsequent SARS-CoV-2 Omicron Infection in Young Children

**DOI:** 10.1001/jamapediatrics.2022.3868

**Published:** 2022-10-24

**Authors:** Alexander C. Dowell, Georgina Ireland, Jianmin Zuo, Paul Moss, Shamez Ladhani

**Affiliations:** 1 Institute of Immunology and Immunotherapy, College of Medical and Dental Sciences, University of Birmingham, United Kingdom; 2 Public Health England, London, United Kingdom

## Abstract

This cohort study investigates the risk of SARS-CoV-2 reinfection among young children with and without spike-specific T-cell responses.

Recent studies highlighted a protective role for preexisting T-cell responses against subsequent SARS-CoV-2 infection in exposed adults.^1,2^ Protection is associated with response against nonstructural,^1,2^ or nonspike, structural proteins.^1^ Responses against spike protein, the immunogen used in most current vaccines, have not been assessed.

The contribution of humoral and cellular responses to vaccine protection remains undefined. Waning antibody titers and variants with enhanced evasion of antibodies, especially Omicron, have raised concerns of a loss of protection. Cellular responses, however, are broadly maintained.^3^ Therefore, the value of spike-specific T-cells in protecting against subsequent infection is of interest.

Previously, in children aged 4 to 11 years, two-thirds were observed to have cellular responses against SARS-CoV-2 spike protein ex vivo in the absence of serologic evidence of previous infection.^4^ This finding provided an opportunity to investigate the association of spike-specific T-cell responses on risk of subsequent SARS-CoV-2 infection in children.

## Methods

This cohort study assessed protection from subsequent SARS-CoV-2 infection in children aged 4 to 11 years in England. The Public Health England Research Ethics and Governance Group approved the study, and written informed consent was obtained. Subsequent symptomatic SARS-CoV-2 infection was assessed through linkage with the national SARS-CoV-2 testing database (Second Generation Surveillance System) until January 31, 2022.

Odds ratios (ORs) with 95% CIs and *P* values were calculated using MedCalc, version 20.115 (MedCalc Software). The study followed STROBE reporting guidelines. The eMethods in the Supplement provide additional details.

## Results

The study included 79 children (mean [SD] age, 7.9 [2.0] years; 39 boys, 37 girls, 3 not recorded; 13 Asian, 8 Black, 38 White, and 20 multiethnic or other ethnicity). A nonsignificant difference was observed in SARS-CoV-2 infection rates in seronegative vs seropositive children with cellular responses (13 of 38 [34%] vs 6 of 37 [16.2%]; OR, 2.69; 95% CI, 0.89-8.08; *P* = .08). A significant difference was found between seronegative children without and seropositive children with a cellular response (7 of 13 [54%] vs 6 of 37 [16.2%]; OR, 6.03; 95% CI, 1.49-24.39; *P* = .01). Seropositive and seronegative children with cellular responses had no significant difference in infection risk (6 of 37 [16.2%] vs 6 of 25 [24%]; OR, 1.63; 95% CI, 0.46-5.80; *P* = .45). Conversely, seronegative children without a cellular response had a higher risk of infection vs those with, but this difference was not significant (7 of 13 [54%] vs 6 of 25 [24%]; OR, 3.69; 95% CI, 0.89-15.37; *P* = .07) (Figure, A). Two of 4 seropositive children without a cellular response had subsequent reinfection, suggesting an association of spike-specific cellular responses with reduced risk of infection (Figure, B). During the Omicron (BA.1) wave (December 1, 2021, to January 31, 2022), infection rates among children with a cellular response were 11% (4 of 35) for seropositive and 5% (1 of 20) for seronegative children vs 45% (5 of 11) for seronegative children without a cellular response (OR, 6.46 [95% CI, 1.33-31.32; *P* = .02] vs 15.83 [95% CI, 1.53-163.56; *P* = .02]) (Figure, C and D).

**Figure.**
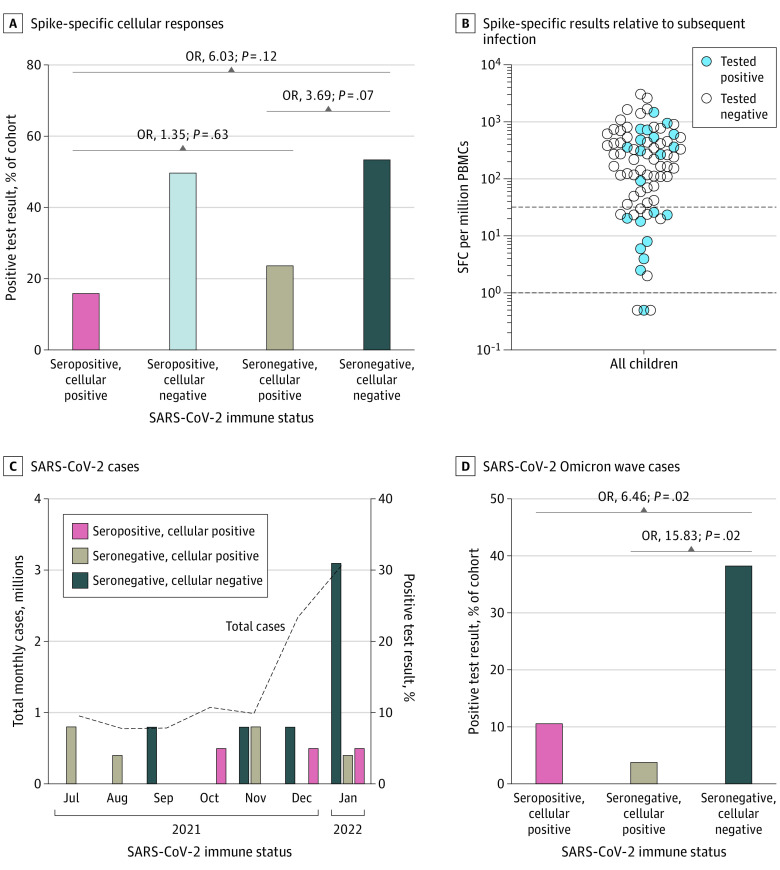
Incidence of SARS-CoV-2 Infection in the Presence of Spike-Specific T Cells in Seronegative Children in England Infection in children confirmed by polymerase chain reaction test; spike-specific cellular responses determined by interferon-γ enzyme-linked immunospot assay. The proportion of the cohort testing positive was calculated as a proportion of the whole cohort at each time point. Children with a positive test in the surveillance period before the SARS-CoV-2 Omicron (BA.1) wave (December 1, 2021, to January 31, 2022) were excluded from analysis of infection during the Omicron wave. PBMC indicates peripheral blood mononuclear cell; SFC, spot-forming cell.

## Discussion

Preexisting cellular responses toward spike protein have not been previously associated with protection. As such, these data may highlight an important role for the cellular response after spike-based vaccines.

That the greatest association was observed during the Omicron wave is important, as previous data showed that the lower-dose BNT162b2 vaccine in children aged 5 to 11 years offers 11% protection from Omicron infection vs 67% in adolescents receiving the standard dose,^5^ despite similar vaccine-induced neutralizing antibody responses in both groups.^6^ Assessing cellular responses in larger cohorts is vital.

This study is limited by the small cohort size. Although cellular responses are associated with protection, addressing the mechanism and role of other innate and mucosal responses is required.

In summary, we found that spike-specific T-cells were associated with relative protection from SARS-CoV-2 infection in young children. This study highlights the importance of assessing T-cell responses as correlates of protection.

## Notes

Supplement.
eMethods
