# Supplementary material for: Association of Spike-Specific T Cells With Relative Protection From Subsequent SARS-CoV-2 Omicron Infection in Young Children
Source: JAMA Pediatr. 2022 Oct 24;177(1):96–7. doi: 10.1001/jamapediatrics.2022.3868 (PMC9593316; doi:10.1001/jamapediatrics.2022.3868)
Supplement: Supplement. — eMethods [file jamapediatr-e223868-s001.pdf]

Supplemental Online Content

Dowell AC, Ireland G, Zuo J, et al. Association of spike-specific T cells with relative protection from subsequent SARS-CoV-2 Omicron infection in young children. *JAMA Pediatr*. Published online October 24, 2022. doi:10.1001/jamapediatrics.2022.3868

eMethods

This supplemental material has been provided by the authors to give readers additional information about their work.

© 2022 American Medical Association. All rights reserved.

eMethods

Sample collection

Public Health England (PHE) initiated prospective SARS-CoV-2 surveillance in primary schools across the UK after they reopened following the easing of national lockdown in June 2020.[1] The protocol for the COVID-19 Surveillance in School KIDs (sKIDs) is available online (https://www.gov.uk/guidance/covid-19-paediatric-surveillance). An initial cohort of seropositive and seronegative children was collected after full reopening of all schools in September 2020, (23 November-18 December), as previously shown.[2] Additional samples were taken from children between 21 June-24 July 2021. No statistical methods were used to pre-determine sample sizes. Researchers were blinded to serostatus of donors prior to ELISpot and serological assessment. Ethical review was provided by the PHE Research Ethics and Governance Group (PHE R&D REGG Ref NR0209). Written informed consent was obtained from all adult participants and from parents or guardians.

All collection and analysis was performed prior to routine vaccination of this age group as such all donors are unvaccinated in the study.

SARS-CoV-2 Infection

We assessed SARS-CoV-2 infection from initial sampling until 31st January 2022, through linkage with the national SARS-CoV-2 testing database (SGSS) held by UK Health Security Agency (UKHSA). SARS-CoV-2 testing was performed following national guidelines. Further details are available from UKHSA online (https://assets.publishing.service.gov.uk/government/uploads/system/uploads/attachment_data/file/926838/PHE_Laboratory_reporting_guidelines_October-2020-v3.pdf).

Omicron Infection

Donors with a positive COVID-19 test between 1st December 2021 and 31st January 2022 were considered to be likely Omicron infections. For analysis of infection rates, donors with a positive test result in the period of follow up until 30th November 2021 were excluded as no data existed as to cellular or serological response following infection; however no omicron re-infection was reported for these recently infected donors.

MSD Serology assay

SARS-CoV-2 serostatus at sampling was determined using the Mesoscale Diagnostics (MSD) platform. Quantitative IgG antibody titres were measured against trimeric spike protein, using MSD V-PLEX COVID-19 Coronavirus Panel 2 (N05368-A1), Multiplex MSD Assays were performed according to

© 2022 American Medical Association. All rights reserved.

manufacturer instructions, as previously described.[2] Spike-specific antibody response was used to define seropositivity using a cut off of 350AU/ml, set in respect of pre-pandemic plasma samples and shown to identify +97% of SARS-CoV-2 PCR-positive children, as previously described.[2]

IFN-γ ELISpot

Briefly, T cell responses were measured against overlapping peptide pools derived from SARS-CoV-2 Spike protein, using a IFN-γ ELISpot Pro kit (Mabtech), cells were plated in duplicate and results were normalised to counts from DMSO negative control wells, and expressed as spot-forming cells per 10^6 input PBMC, as previously described.[2] A Cut-off value of 32 sfc/10^6 PBMC (median+2SD) was previously defined as a positive spike response determined using pre-pandemic samples from 17 adults.[3] Comparison of responses in adults and children are shown in the previous pubblication.[2]

Reporting guidelines

This report followed STROBE guidelines for reporting cohort studies.

Statistical calculations

Odds ratios (OR) and associated p-values were calculated using MedCalc[4]. OR are shown including 95% confidence intervals.

1. Ladhani SN, Baawuah F, Beckmann J, et al. SARS-CoV-2 infection and transmission in primary schools in England in June-December, 2020 (sKIDs): an active, prospective surveillance study. *Lancet Child Adolesc Health.* 2021;5(6):417-427.
2. Dowell AC, Butler MS, Jinks E, et al. Children develop robust and sustained cross-reactive spike-specific immune responses to SARS-CoV-2 infection. *Nature Immunology.* 2022;23(1):40-49.
3. Zuo J, Dowell AC, Pearce H, et al. Robust SARS-CoV-2-specific T cell immunity is maintained at 6 months following primary infection. *Nature Immunology.* 2021;22(5):620-626.
4. MedCalc Statistical Software. MedCalc Software Ltd., Ostend, Belgium. https://www.medcalc.org/calc/odds_ratio.php. Published 2020. Accessed 01/02/2022, 2022.

© 2022 American Medical Association. All rights reserved.
